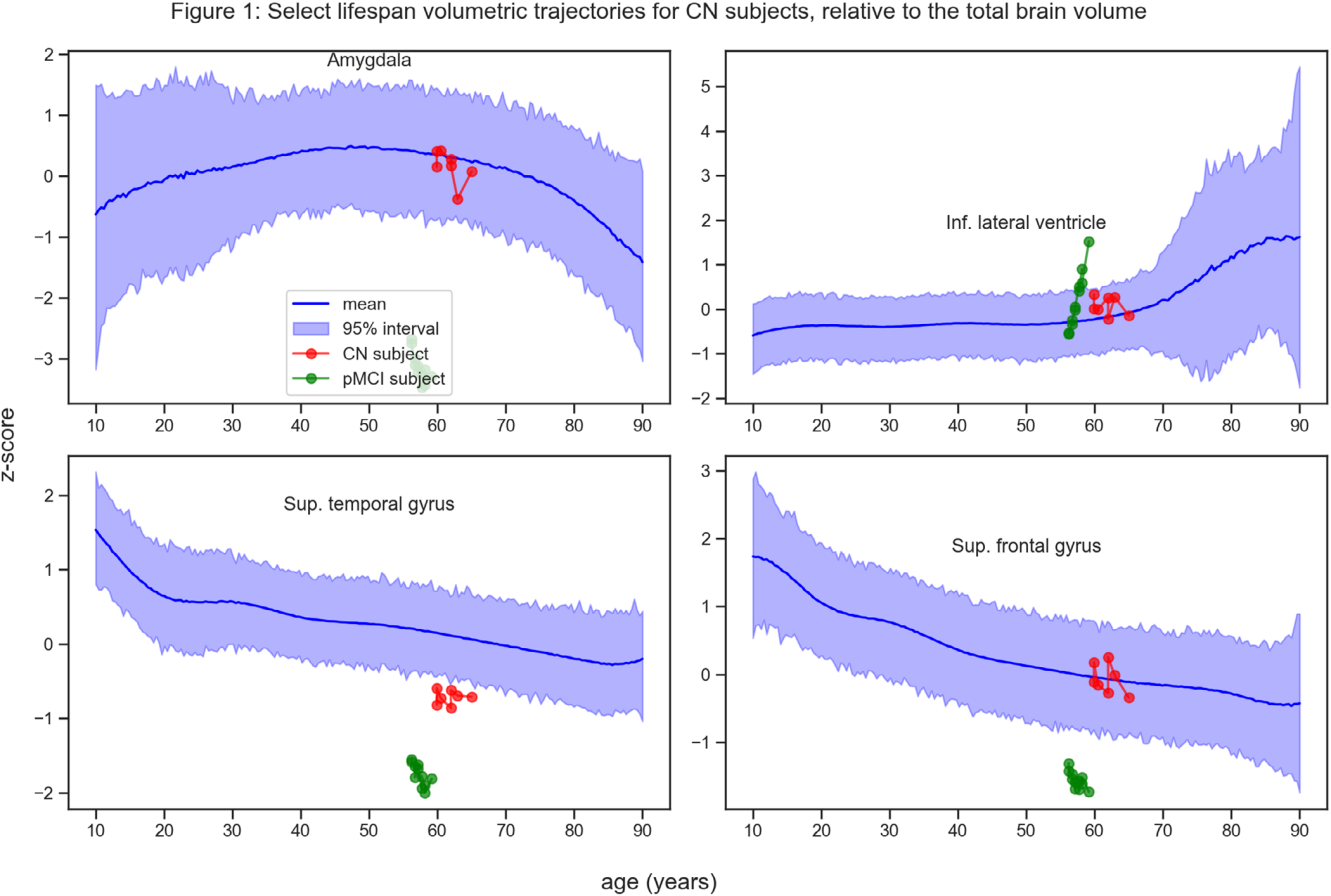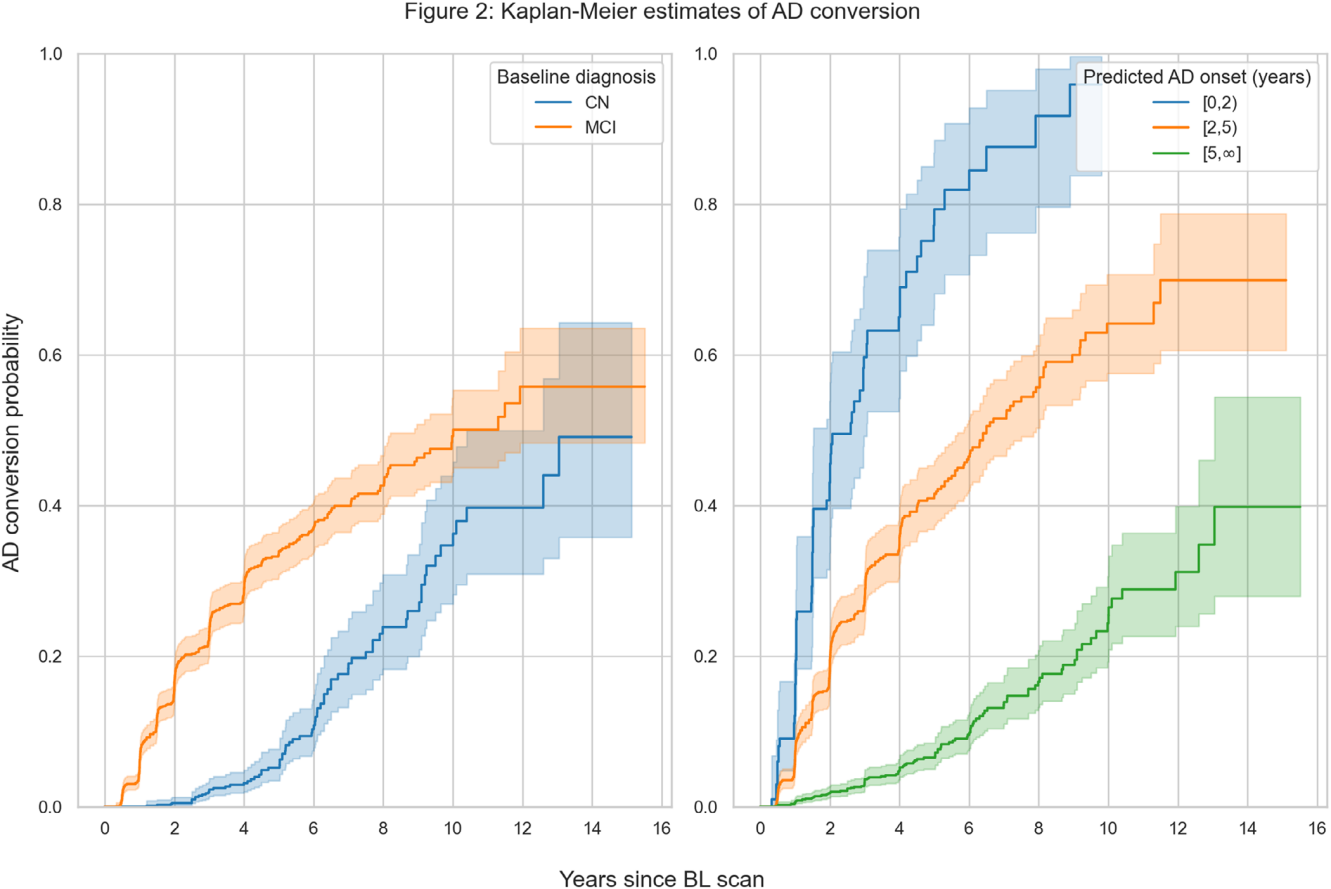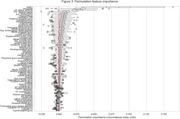# Patient‐level predicting AD onset using lifespan volumetric trajectories

**DOI:** 10.1002/alz70856_107271

**Published:** 2026-01-09

**Authors:** Vladimir S Fonov, Pedro Rosa‐Neto, D Louis Collins

**Affiliations:** ^1^ McConnell Brain Imaging Centre, Montreal Neurological Institute, McGill University, Montreal, QC, Canada; ^2^ Translational Neuroimaging Laboratory, Montreal, QC, Canada; ^3^ Montreal Neurological Institute, Montreal, QC, Canada

## Abstract

**Background:**

Predicting dementia risk from neuroimaging and cognitive data is vital for early Alzheimer's disease (AD) management. Coupe [2019] and others have shown that the volume of certain anatomical structures deviate from the normal trajectories. We aim to predict individual AD progression risk by analyzing deviations from expected age and sex‐based volumetric measurements at the baseline.

**Method:**

We utilized T1w MRI scans from several databases: (*N* Scans, N subjects, age range) : ADNI1,2,3 (8697, 2118, 50‐97y), AIBL (1242, 667, 55‐97y), HCP (1113, 1113, 28‐36y), ICBM (341, 341, 18‐80y), MCSA (1801,1801, 49‐89y), NIHPD (1089, 442,4‐22y), NKI (2306, 1326, 6‐85y), OASIS1,2,3 (3212, 1802, 18‐97y), UK Biobank (47396, 42912, 44‐83y), PreventAD (2400, 387, 54‐88y) and TRIAD (914,20‐91y) and processed them with AssemblyNET [Coupe 2019].

We used scans from UKBB, ICBM, NKI, HCP, and NIHPD studies, along with half of the cognitively normal (CN) subjects from ADNI1,2,3, to model healthy aging trajectories with cubic b‐splines for each brain structure from ages 10 to 90 using a Bayesian multilevel model.

We used baseline scans from remaining CN subjects (*N* = 984, 73 progressed to AD) and MCI subjects (*N* = 1396, 395 progressed) to calculate deviation from age and sex expected volumes as estimated for 70 ROIs extracted by AssemblyNET. These values, along with sex and baseline diagnosis, were used to perform time‐to‐event modelling of conversion to AD. We employed a linear Survival Support Vector Machine within a 10‐fold cross‐validation loop. Additionally, permutation importance was used to identify important ROIs.

**Result:**

Our results demonstrate that the proposed method effectively identifies individuals with varying levels of risk for AD conversion, achieving a concordance index of 0.82(0.04). The five most important features identified by the method are baseline diagnosis (CN or MCI), and volumes of Amygdala, Inferior lateral ventricles, Superior temporal gyrus and Superior frontal gyrus.

**Conclusion:**

We have developed a library of healthy aging trajectories for 70 anatomical structures, demonstrating its effectiveness in identifying subjects at high risk of progressing to Alzheimer's Dementia.